# Post‐endoscopic sphincterotomy delayed bleeding occurs in patients with just 1‐day interruption of direct oral anticoagulants or hemodialysis

**DOI:** 10.1002/deo2.70060

**Published:** 2025-01-16

**Authors:** Sho Hasegawa, Yusuke Kurita, Yuma Yamazaki, Shinichi Nihei, Takeshi Iizuka, Noboru Misawa, Kunihiro Hosono, Itaru Endo, Noritoshi Kobayashi, Kensuke Kubota, Atsushi Nakajima

**Affiliations:** ^1^ Department of Gastroenterology and Hepatology Yokohama City University Graduate School of Medicine Kanagawa Japan; ^2^ Department of Gastroenterological Surgery Yokohama City University Graduate School of Medicine Kanagawa Japan; ^3^ Department of Oncology Yokohama City University Graduate School of Medicine Kanagawa Japan

**Keywords:** bleeding, dialysis, endoscopic sphincterotomy, ERCP, oral anticoagulant

## Abstract

**Objective:**

Endoscopic sphincterotomy (EST), especially when anticoagulants are used, carries a significant risk of delayed bleeding. However, the relationship between the use of antithrombotic agents, including direct oral anticoagulants, and post‐EST bleeding remains unclear. This study aimed to identify the risk factors for post‐EST delayed bleeding when antithrombotic agents were administered according to the guidelines.

**Methods:**

We analyzed cases of patients who underwent endoscopic retrograde cholangiopancreatography and EST between January 2018 and August 2022, focusing on those with normal anatomy and naïve papillae. We examined the incidence of post‐EST bleeding, endoscopic retrograde cholangiopancreatography procedure details, severity and timing of post‐EST delayed bleeding, hemostatic interventions, and factors related to post‐EST delayed bleeding.

**Results:**

Among the 502 patients included, 76 (15%) were taking antithrombotic agents. Post‐endoscopic retrograde cholangiopancreatography delayed bleeding was noted in seven patients (1.4%). Mild, moderate, and severe delayed bleeding occurred in four, one, and two cases, respectively. Hemostatic injection completely controlled cases of delayed bleeding. Multivariate analysis identified a 1‐day direct oral anticoagulants interruption (odds ratio: 20.5, 95% confidence interval: 3.33–125, *p* = 0.0011) and dialysis (odds ratio: 38.7, 95% confidence interval: 2.4–624, *p* = 0.0099) as significant risk factors for delayed bleeding. No thromboembolic events related to the discontinuation of antithrombotic drugs were observed.

**Conclusion:**

A 1‐day direct oral anticoagulants interruption and dialysis are independent risk factors for post‐EST delayed bleeding, necessitating careful consideration.

## INTRODUCTION

Endoscopic sphincterotomy (EST) is a basic and established technique used in endoscopic retrograde cholangiopancreatography (ERCP), the utility of which is well established. However, it is associated with a high risk of bleeding, including intraoperative and delayed bleeding. Delayed bleeding can lead to major hemorrhage and often poses marked clinical challenges.^1^ It is reported to occur at a frequency of 1–2%,^2^, ^3^, ^4^ and risk factors include dialysis and heparin bridging for warfarin.^2^, ^5^ In recent years, management with antithrombotic agents, including direct oral anticoagulants (DOACs), has been established according to the Japan Gastroenterological Endoscopy Society (JGES).^6^, ^7^ Since patients receiving antithrombotic therapy have a higher risk of bleeding, the guidelines of the European Society of Gastrointestinal Endoscopy, American Society for Gastrointestinal Endoscopy, and Asian Pacific Association of Gastroenterology recommend longer interruption periods for DOACs than that recommended by the JGES.^8^, ^9^, ^10^ However, evidence remains weak, and the fragile relationship between the interruption period and the risk of post‐EST bleeding remains a topic of debate. DOACs are promising in presenting a lower risk of gastrointestinal bleeding compared to warfarin^11^; however, there are few reports on post‐EST bleeding following guideline‐based management with DOACs.

Regarding the antiplatelet agents used during endoscopic treatment, continuation of aspirin monotherapy is recommended considering the embolism risk, because it does not exacerbate bleeding.^4^, ^12^, ^13^, ^14^ However, reports suggesting that the continuation of antiplatelet monotherapy increases the risk of post‐EST bleeding have made this issue controversial.^15^ At present, the relationship between antithrombotic management according to the JGES guidelines and post‐EST bleeding remains unclear. Hence, this study aimed to identify the risk factors for post‐EST bleeding when antithrombotic agents are used according to the JGES guidelines.

## METHODS

### Patients

This study was approved by the Ethics Committee of Yokohama City University (F221100031). Data of patients who underwent ERCP at Yokohama City University Hospital between January 2018 and August 2022 were reviewed, and those of 502 patients with normal anatomy and untreated papillae who underwent EST were analyzed. The procedures described were performed in accordance with the ethical principles of the Declaration of Helsinki. Informed consent was obtained from all participants by using an opt‐out approach.

### Endoscopic procedure

In all cases, ERCP was performed under fluoroscopic guidance. A JF‐260 V or TJF‐260 V scope was used, and sedation was achieved using pentazocine, midazolam, diazepam, or propofol when needed. EST was performed after bile duct cannulation and cholangiography using Clever Cut 3 V (Olympus Medical Systems). The electrosurgical unit used during EST was the ICC 200, which was set to 120 W in the Endocut mode. EST was performed in combination with endoscopic papillary large balloon dilatation (EPLBD) as needed. Bile duct stone extraction or biliary stent placement was performed depending on the case. Stone removal was performed using a mechanical lithotripter, basket, or balloon catheter. For the removal of large stones, electrohydraulic lithotripsy under cholangioscopy was used in combination with the aforementioned measure. For bile duct strictures, balloon dilation, plastic or metal stent placement, or endoscopic nasobiliary drainage (ENBD) was performed. Endoscopic hemostasis was achieved using local injection of hypertonic saline epinephrine (HSE), thrombin application, and clipping.

### Anticoagulant drug management

Based on the Guidelines for Gastroenterological Endoscopy in Patients Undergoing Antithrombotic Treatment,^6^, ^7^ instructions for medication were provided for EST because the procedure is associated with a high risk of bleeding. Regarding antiplatelet agent use, EST was performed without interruption if the patient was on only aspirin or cilostazol. If the patient was receiving clopidogrel, it was replaced with aspirin or cilostazol before the procedure. DOACs such as dabigatran, rivaroxaban, apixaban, and edoxaban were discontinued on the morning of the ERCP, and EST was performed, with medication reinitiation on the following morning. The resumption of DOAC administration was postponed in cases where overt bleeding or worsening anemia, suggestive of post‐EST bleeding, was observed. If the patient was on warfarin, a temporary switch to DOACs was made if possible; if not, warfarin was discontinued and heparin replacement was used. Heparin was discontinued on the morning of the EST day and restarted 2 h after the procedure. EST was not performed in cases where the discontinuation of two or more antiplatelet agents or a combination of antiplatelet agents and DOACs was not feasible. In dialysis patients, nafamostat mesylate was used for hemodialysis during the hospitalization period for ERCP, while heparin was used after discharge.

### Definition of bleeding

**FIGURE 1:**
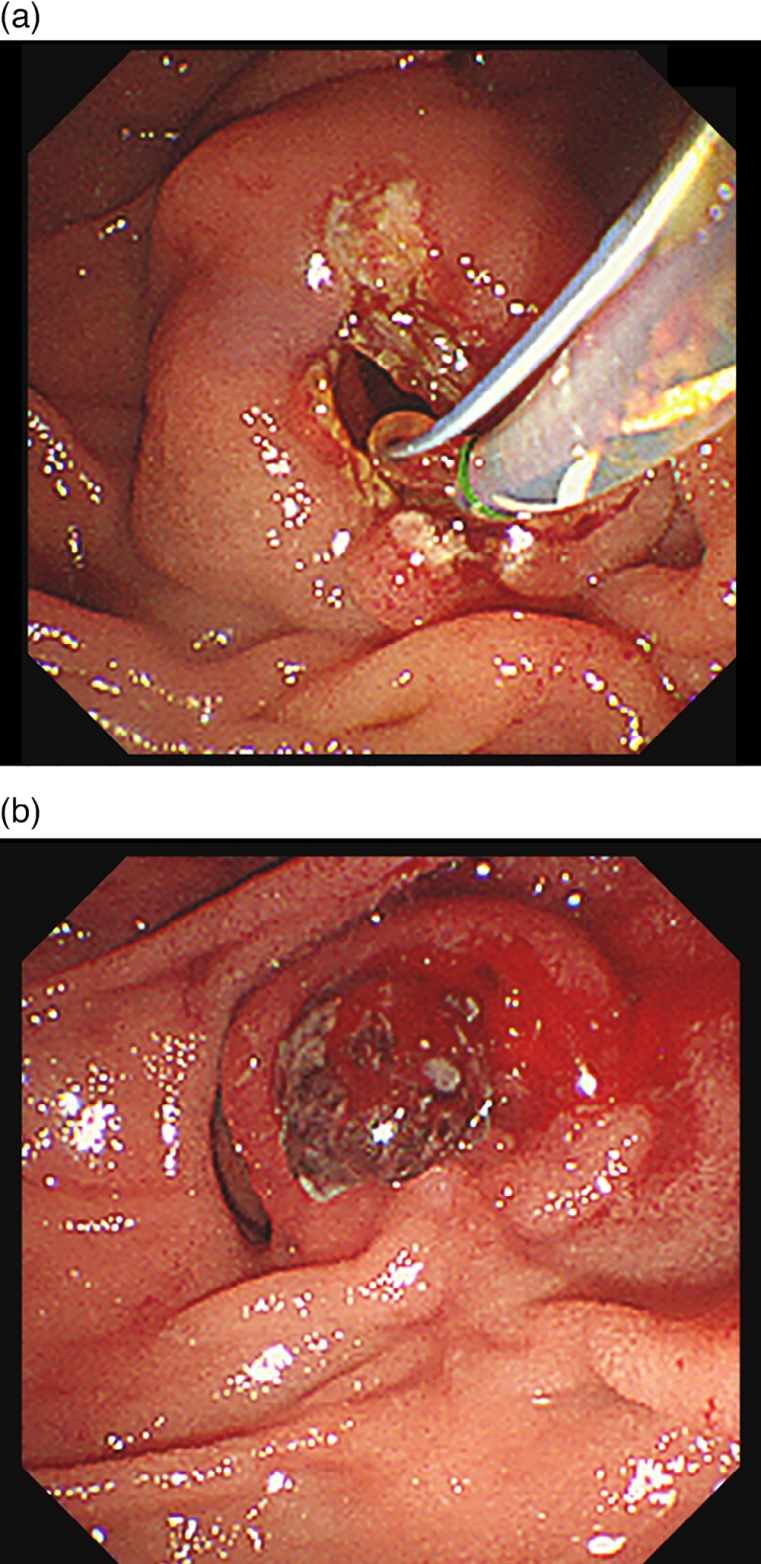
Endoscopic images of post‐EST bleeding. (a) Papilla during EST. (b) Papilla 2 days after EST, showing evidence of bleeding. EST: endoscopic sphincterotomy.

ERCP‐related bleeding was classified as immediate or delayed. Immediate bleeding was defined as active bleeding requiring hemostatic treatment during ERCP or within 24 h after the procedure. Delayed bleeding was defined as a decrease of ≥2 g/dL in the hemoglobin level occurring 24 h or later, accompanied by overt signs, such as hematemesis or melena, or symptoms of circulatory failure due to anemia, requiring second‐look endoscopy (Figure 1). Bleeding severity was classified into three grades using the Cotton classification: mild, moderate, and severe.^16^

### Outcomes

The primary outcome was the incidence of post‐EST bleeding. The incidence of intraoperative bleeding during EST, the details of the ERCP procedure, severity and timing of post‐EST delayed bleeding, hemostatic methods, and factors associated with post‐EST delayed bleeding were also analyzed.

### Statistical analysis

Categorical variables were compared using the chi‐squared test or Fisher's exact test. For continuous data, the Student's *t*‐test or Mann‐Whitney *U* test was used. Multivariate logistic regression analysis was performed. Statistical significance was set at *p* < 0.05. Statistical analyses were performed using JMP Pro (version 16.0; SAS Institute, Inc.).

## RESULTS

### Patient characteristics

Among the 502 patients, 299 were male (59%) and the median patient age was 72 years. The primary conditions were bile duct stones, pancreatic cancer, bile duct cancer, benign bile duct strictures, and acute cholecystitis in 209 (42%), 121 (24%), 106 (21%), 34 (6.7%), and 10 (2.4%) patients, respectively. Comorbidities included cardiovascular disease, intracerebral hemorrhage, cerebral infarction, Child‐Pugh C liver cirrhosis, chronic kidney failure on maintenance dialysis, and cholangitis in 81 (16%), 22 (4.3%), eight (1.6%), seven (1.3%), five (1%), and 139 (27%) patients, respectively. Antithrombotic drug use was observed in 76 patients (15%): 45 (8.9%), 26 (5.1%), and nine (1.7%) patients were on single antiplatelet therapy, DOACs, and warfarin with heparin bridging, respectively. No cases of coagulation abnormalities that would preclude EST were identified before ERCP (Table 1).

**TABLE 1:** Patient characteristics.

*n*	502
Sex, male, *n* (%)	299 (59)
Age, median (range)	72 (20–93)
Primary disease, *n* (%)	
Bile duct stone	209 (42)
Pancreatic cancer	121 (24)
Bile duct cancer	106 (21)
Benign biliary structure	34 (6.7)
Lymph node metastasis of other cancers	22 (4.1)
Cholecystitis	10 (2.4)
Underlying disease	
Cardiovascular disease	81 (16)
Cerebral hemorrhage	22 (4.3)
Stroke	8 (1.6)
Liver cirrhosis	7 (1.3)
Hemodialysis	5 (1)
Cholangitis	139 (27)
Antithrombotic medications	76 (15)
Antiplatelet agents	45 (8.9)
DOACs	26 (5.1)
Heparin bridging of warfarin	9 (1.7)
PT‐INR (IQR)	1.07 (1.02–1.15)
APTT, second (IQR)	32.1 (29.8–34.5)
Platelet count, /µL (IQR)	21.2 (16.6–26.3)
eGFR	73.3 (59.7–88.3)

Abbreviations: APTT, activated partial thromboplastin time; DOAC, direct oral anticoagulant; eGFR, estimated glomerular filtration rate; PT‐INR, prothrombin time‐international normalized ratio.

### ERCP procedure and complications

ERCP procedures included biliary drainage, stone removal, and diagnosis only in 311 (62%), 175 (34%), and 22 (4%) cases, respectively. The biliary drainage involved the use of plastic stents, self‐expandable metal stents (SEMS), and ENBD in 172 (34%), 113 (20%), and 26 (5%) cases, respectively. The periampullary diverticulum was observed in 92 cases (18%). The incision size for EST was small in 435 cases (87%) and medium in 76 cases (13%), with no cases requiring a large incision. EST was performed in combination with EPLBD in 31 cases (6.1%). The average procedure time for ERCP (range) was 30 min (range; 7–120 min). Intraoperative bleeding occurred in 44 patients (8.7%). Other complications included post‐ERCP pancreatitis, cholecystitis, and cholangitis in 70 (14%), nine (1.7%), and seven (1.4%) cases, respectively. Of the post‐ERCP pancreatitis cases, 51 (10%), 11 (2%), and 8 (1.5%) were mild, moderate, and severe, respectively (Table 2). No thromboembolic events related to the discontinuation of antithrombotic drugs were observed.

**TABLE 2:** Endoscopic retrograde cholangiopancreatographyprocedure and complications.

Purpose	*n* (%)
Stone removal	175 (34)
Biliary drainage	
Plastic stent	172 (34)
SEMS	107 (21)
ENBD	26 (5)
Diagnosis, biopsy	22 (4)
Procedure time, min (range)	30 (7–120)
Periampullary diverticulum, *n* (%)	92 (18)
Extent of the EST incision, *n* (%)	
Small	435 (87)
Medium	67 (13)
Full	0 (0)
Combination with EPLBD, *n* (%)	31 (6.1)
Adverse event without post‐EST delayed bleeding, *n* (%)	
Intraoperative bleeding	44 (8.7)
Pancreatitis	70 (14)
Mild	51 (10)
Moderate	11 (2)
Severe	8 (1.5)
Cholecystitis	9 (1.7)
Cholangitis	7 (1.4)
Perforation	0 (0)

Abbreviations: ENBD, endoscopic nasobiliary drainage; EPLBD, endoscopic papillary large balloon dilatation.; ERCP, endoscopic retrograde cholangiopancreatography; EST, endoscopic sphincterotomy; SEMS, self‐expandable metallic stent.

### Incidence, timing, and management of post‐EST delayed bleeding

Delayed bleeding after ERCP occurred in seven patients (1.4%). Delayed bleeding occurred at the following time points: between 24 and 48 h, two cases; between 48 and 72 h, two cases; and after 72 h, three cases. The severity of bleeding was mild, moderate, and severe in four, one, and two cases, respectively. All cases of delayed bleeding were managed using HSE injections for primary hemostasis, and clipping was performed in only one case (Table 3).

**TABLE 3:** Details of post‐endoscopic sphincterotomy delayed bleeding.

Post‐EST delayed bleeding	
Incidence, *n* (%)	7 (1.4)
Time to diagnose, median (range)	
24–48 h	2
48–72 h	2
>72 h	3
Severity grading	
Mild	4
Moderate	1
Severe	2
Method of hemostasis	
HSE injection	7
Clipping	1

Abbreviations: ERCP, endoscopic retrograde cholangiopancreatography; EST, endoscopic sphincterotomy; HSE, hypertonic saline epinephrine solution.

**TABLE 4:** Multivariate analysis of factors associated with post‐endoscopic sphincterotomy delayed bleeding.

	Bleeding (*n* = 7)	No bleeding (*n* = 495)	Univariate analysis, *p*‐value	Multivariate analysis, *p*‐value	Odds ratio	95% CI
Age, >75 years	4 (57%)	207 (41%)	0.4610			
Malignant disease	2 (29%)	247 (50%)	0.4499			
Cholangitis	1 (14%)	138 (28%)	0.6794			
Periampullary diverticulum	3 (43%)	89 (18%)	0.1191	0.5401	1.7	0.29–10.0
Median incision range for EST	2(28%)	65 (13%)	0.2371			
Combination with EPLBD	2 (28%)	29 (5.8%)	0.0639	0.0662	5.7	0.88–37.1
Biliary stenting	5 (71%)	300 (61%)	0.7098			
SEMS replacement	0 (0%)	107 (21%)	0.3547			
During bleeding	2 (29%)	42 (8.4%)	0.1189	0.0559	6.4	0.92–45.2
PT‐INR >1.5	1 (14%)	17 (3.4%)	0.2268			
Antiplatelet agent	0 (0%)	45 (9%)	1.0000			
DOACs	3 (43%)	23 (4.6%)	**0.0038**	**0.0011**	20.5	3.33–125
Heparin bridging of warfarin	0 (0%)	9 (1.8%)	1.0000			
Liver cirrhosis	0 (0%)	1 (0.2%)	0.115			
Hemodialysis	1 (14%)	4 (0.8%)	0.0681	**0.0099**	38.7	2.4–624

Abbreviations: DOAC, direct oral anticoagulant.; EPLBD, endoscopic papillary large balloon dilatation; ERCP, endoscopic retrograde cholangiopancreatography; EST, endoscopic sphincterotomy; PT‐INR, prothrombin time‐international normalized ratio; SEMS, self‐expandable metallic stent.

### Factors related to delayed bleeding

Univariate analysis identified DOAC use as a significant risk factor for delayed bleeding among factors such as age (75 years or older), malignant disease, presence of cholangitis or periampullary diverticulum, medium‐sized incision EST, EST performed in combination with EPLBD, bile duct stent placement, intraoperative bleeding, continuation of antiplatelet therapy, 1‐day DOAC interruption, heparin bridging, liver cirrhosis (Child‐Pugh C), and dialysis (*p* = 0.0038). Multivariate analysis using ERCP‐related bleeding, 1‐day DOAC interruption, and dialysis as variables identified 1‐day DOAC interruption (odds ratio [OR]: 20.5, 95% confidence interval [CI]: 3.33–125, *p* = 0.0011) and dialysis (OR: 38.7, 95% CI: 2.4–624, *p* = 0.009) as significant risk factors for delayed bleeding (Table 4).

## DISCUSSION

In this study, we evaluated the data of patients who underwent ERCP according to the JGES guidelines for management with antithrombotic drugs and examined the results related to post‐EST delayed bleeding. We found that a 1‐day interruption of DOACs was a significant risk factor for post‐EST delayed bleeding. Receipt of maintenance dialysis was also identified as a significant risk factor. In contrast, the continuation of single antiplatelet therapy was not a risk factor for delayed bleeding. Thromboembolic events were not observed in the patients studied.

EST is a high‐risk procedure, and post‐EST bleeding presents clinical challenges. In this study, the frequency of post‐EST bleeding was 1.4%, which is consistent with previously reported rates of 1%–4%,^2^, ^17^, ^18^, ^19^ indicating that the post‐EST bleeding rate in this study is reasonable. The responses to post‐EST bleeding included clipping, HSE injection, balloon tamponade, and argon plasma coagulation. In all cases, primary hemostasis was achieved using HSE injections, with clipping performed in one case involving a dialysis patient. None of the patients required arterial embolization or surgery. Since post‐EST bleeding can sometimes be significant and present clinical issues, it is essential to remain vigilant.

A 1‐day interruption of DOACs was identified as a significant risk factor for post‐EST bleeding. Given that DOACs have a short half‐life of approximately 12 h, a 1‐day interruption before EST, as per the JGES guidelines, is considered reasonable. However, because peak plasma concentrations of DOACs are reached within 1–3 h after administration,^20^ resuming medication could lead to a sudden increase in bleeding tendency and potentially trigger delayed bleeding. In our study, the resumption of DOACs on the day after EST was associated with subsequent bleeding. Guidelines on DOAC management during EST vary among countries. The American Society for Gastrointestinal Endoscopy and the European Society of Gastrointestinal Endoscopy recommends resuming medication 1–3 and 2–3 days after the procedure, respectively, while the JGES recommends resumption on the day after the procedure. Japanese reports suggest that an interruption of more than 1‐day results in fewer post‐EST bleeding events than an interruption of 1 day.^19^ Furthermore, meta‐analyses of antithrombotic drug use showed that a 1‐day interruption did not significantly reduce the bleeding risk.^21^ Although Western guidelines consider bleeding risks when setting interruption periods, there is also the risk of thromboembolic events during the interruption period. In this study, because all cases involved a 1‐day interruption, the absence of thromboembolic events suggests that the JGES guidelines are acceptable. However, because this study found that a 1‐day interruption of DOACs was a significant risk factor for delayed bleeding, further investigation into the optimal timing for resuming DOACs is needed. For patients with a low risk of embolism, delaying the resumption of DOACs after EST might be considered.

In recent years, the utility of combining small EST with EPBD (ESBD) or with EPLBD (ESLBD) has been reported.^22^ ESBD for small stones is associated with a significantly lower incidence of bleeding compared with EST,^23^ suggesting it may be a safer option for patients with a bleeding tendency. On the other hand, for large stones, ESLBD has been reported to be associated with a similar incidence of bleeding as EST.^24^

In this study, only 6.1% of cases underwent ESLBD, and it was not identified as a risk factor for either intraoperative or post‐EST delayed bleeding. For patients with a tendency to bleed, ESBD or ESLBD should be considered as potential options in future clinical practice.

Reports have indicated that hemodialysis in patients with chronic renal failure is a risk factor for post‐EST bleeding.^2^, ^5^, ^25^, ^26^ Multivariate analysis in this study also identified hemodialysis as a significant risk factor for post‐EST bleeding, consistent with previous reports. Patients undergoing hemodialysis for chronic renal failure are believed to have higher bleeding tendencies owing to impaired platelet function and intermittent anticoagulant use. Although only five patients on hemodialysis were included in this study, it is essential to consider the increased risk of post‐EST bleeding when performing EST in such patients.

However, continued use of aspirin monotherapy was not identified as a risk factor for post‐EST delayed bleeding. Although some meta‐analyses have shown that aspirin increases the risk of post‐EST bleeding,^27^ there are also reports indicating that aspirin monotherapy does not significantly increase bleeding risk.^4^, ^12^, ^13^ Various guidelines indicate that aspirin therapy can be continued during EST.^6^, ^8^, ^10^ The results of this study support the safety of continued aspirin therapy during EST according to the guidelines.

Intraoperative bleeding during EST was not a significant risk factor for post‐EST delayed bleeding in this study. However, intraoperative bleeding has been reported as a risk factor for post‐EST bleeding in a large retrospective study.^18^ In the present study, too, multivariate analysis revealed that intraoperative bleeding tended to be a risk factor for post‐EST bleeding (OR = 6.4; 95% CI: 0.92–45.2; *p* = 0.0559). If more cases are identified, it may be extracted as a significant risk factor, and patients with intraoperative bleeding during EST should be cautioned against post‐EST bleeding as well. We also analyzed risk factors associated with intraoperative bleeding during EST (Table S1) and found that 1‐day withdrawal of DOACs and maintenance dialysis, which were identified as risk factors for post‐EST bleeding, were not risk factors for intraoperative bleeding. They were also not associated with the use of antiplatelet agents. Therefore, we believe that intraoperative bleeding can be safely treated with antithrombotic agents according to the guidelines.

This study has several limitations. One limitation is that it is a single‐center, retrospective analysis, which introduces the possibility of selection bias. Therefore, large‐scale validation across multiple centers is required. Additionally, post‐EST bleeding is a relatively rare complication compared with ERCP‐related pancreatitis, resulting in a low incidence rate.

In conclusion, 1‐day interruption of DOACs and hemodialysis are independent risk factors for post‐EST bleeding. For patients with a low risk of embolism, delaying the resumption of DOACs after EST might be considered.

## CONFLICT OF INTEREST STATEMENT

None.

## ETHICS STATEMENT

Approval of the research protocol by an Institutional Reviewer Board: The study was approved by the ethics committee of Yokohama City University (F221100031).

## PATIENT CONSENT STATEMENT

Informed consent was obtained from all participants by using an opt‐out approach.

## CLINICAL TRIAL REGISTRATION

N/A.

## Supporting information

Supplemental Table 1. Multivariate analysis of the factors associated with intraoperative bleeding during EST.
