# Supplementary material for: Post‐endoscopic sphincterotomy delayed bleeding occurs in patients with just 1‐day interruption of direct oral anticoagulants or hemodialysis
Source: DEN Open. 2025 Jan 16;5(1):e70060. doi: 10.1002/deo2.70060 (PMC11736286; doi:10.1002/deo2.70060)
Supplement: Supplementary file 1 — Supplemental Table 1. Multivariate analysis of the factors associated with intraoperative bleeding during EST. [file DEO2-5-e70060-s001.docx]

**Supplemental Table 1. Multivariate analysis of the factors associated with intraoperative bleeding during EST.**

|  | Bleeding  (n = 44) | No bleeding  (n = 458) | Univariate analysis, P-value | Multivariate analysis, P-value | Odds ratio | 95% CI |
| --- | --- | --- | --- | --- | --- | --- |
| Periampullary diverticulum | 9 (20%) | 83 (18%) | 0.7025 |  |  |  |
| Median incision range for EST | 9 (20%) | 58 (13%) | 0.1466 | 0.2632 | 1.6 | 0.7-3.5 |
| Combination with EPLBD | 3 (6.8%) | 28 (6.1%) | 0.8529 |  |  |  |
| Biliary stenting | 14 (31%) | 291 (63%) | **<0.0001** | **0.0001** | **0.27** | **0.13-0.52** |
| SEMS replacement | 5 (11%) | 102 (22%) | 0.1215 |  |  |  |
| PT-INR > 1.5 | 1 (2.3%) | 17 (3.7%) | 0.6239 |  |  |  |
| Antiplatelet agent | 6 (14%) | 39 (8.5%) | 0.2560 | 0.3109 | 1.6 | 0.63-4.27 |
| DOACs | 1 (2.3%) | 25 (5.4%) | 0.7175 | 0.3796 | 0.4 | 0.05-3.01 |
| Heparin bridging of warfarin | 0 (0%) | 9 (1.9%) | 1.0000 |  |  |  |
| Liver cirrhosis | 0 (0%) | 7 (1.5%) | 1.0000 |  |  |  |
| Hemodialysis | 1 (2.2%) | 4 (0.9%) | 0.3691 | 0.4558 | 2.4 | 0.32-19.2 |
|  |  |  |  |  |  |  |

Abbreviations: EST, endoscopic retrograde cholangiopancreatography; EPLBD, endoscopic papillary large balloon dilatation; SEMS, self-expandable metallic stent; PT-INR, prothrombin time-international normalized ratio; DOAC, direct oral anticoagulant.
